# Ruptured splenic abscess as an extremely rare cause of pneumoperitoneum: A comprehensive review with a case report

**DOI:** 10.1016/j.radcr.2023.09.025

**Published:** 2023-10-04

**Authors:** Elham Zarei, Arash Pour Mohammad, Mehdi Vafadar, Milad Gholizadeh Mesgarha

**Affiliations:** a Department of Radiology, Ali Asghar Children Hospital, School of Medicine, Iran University of Medical Sciences (IUMS), Tehran, Iran; b Faculty of Medicine, Iran University of Medical Sciences (IUMS), Tehran, Iran; c Department of Pediatric Endocrinology, Ali Asghar Children Hospital, School of Medicine, Iran University of Medical Sciences (IUMS), Tehran, Iran

**Keywords:** Splenic abscess, Pneumoperitoneum, Splenic perforation, Diabetes mellitus

## Abstract

Splenic abscess leading to rupture and pneumoperitoneum is a very rare condition that is limited to a few case reports. In this study, we first introduced a case of an adolescent girl with a medical history of diabetes mellitus who presented with left upper quadrant abdominal pain and fever. Ultrasound revealed an abscess in the spleen and a computed tomography (CT) scan of the abdomen revealed evidence of pneumoperitoneum secondary to the rupture of this abscess. The patient underwent splenectomy without postoperative complications.

Secondly, we reviewed and discussed the current literature on this topic which predominantly denotes that the pneumoperitoneum following splenic abscess rupture mostly occurs in the immunocompromised status, without a specific predominant infectious agent but the culprit is a gas-forming organism, acute abdomen ensues if the diagnosis is delayed, diagnosis is via abdominal CT when there is hemodynamic stability otherwise exploratory laparotomy uncovers the diagnosis, and splenectomy with broad-spectrum antibiotic therapy is the mainstay of treatment.

## Introduction

Splenic abscess is an infrequent but potentially life-threatening disease [1]. It could give rise to some fairly common complications such as left-sided pleural effusion and splenomegaly but limited to very few case reports, splenic abscess could rupture and lead to pneumoperitoneum which subsequently cause peritonitis and acute abdomen [2,3]. This extremely rare condition primarily manifests in immunocompromised settings, whereby a patient exhibiting characteristic signs of splenic abscesses, such as left upper quadrant pain and fever, then has generalized abdomen pain and tenderness after an interval of inadequate therapeutic intervention [3,4].

Herein, we report a case of pneumoperitoneum that occurred following perforation of a splenic abscess in an adolescent girl with a medical history of diabetes mellitus and then discuss and review reported cases in this subject area. To the best of our knowledge, this is the first case in the literature that describes this extremely rare complication in the pediatric population.

## Case presentation

### History and physical examination

A 14-year-old female patient was admitted to the emergency department suffering from abdominal pain and fever. Her symptoms began approximately 2 weeks before presentation when she developed progressive abdominal pain in the left upper quadrant (LUQ) region. She also reported having intermittent low-grade fever during her disease course. According to these symptoms, she received supportive treatment with a presumptive diagnosis of transient viral infection. However, her symptoms did not abate and her pain intensity increased and was associated with constant fever resulting in her hospitalization. Her medical history was notable for type I diabetes mellitus since 5 years ago and vesicourethral reflux but with no recent urinary tract infection. There was also no history of abdominal trauma, sickle cell anemia, malignancy, or use of immunosuppressive medications. On physical examination, a temperature of 38.4°C was noted, and vital signs were otherwise normal. Diminished lung sound was detected at the base of the left lung and LUQ tenderness, and splenomegaly were noticed on abdominal examination.

### Paraclinical tests and diagnosis

**Fig. 1:**
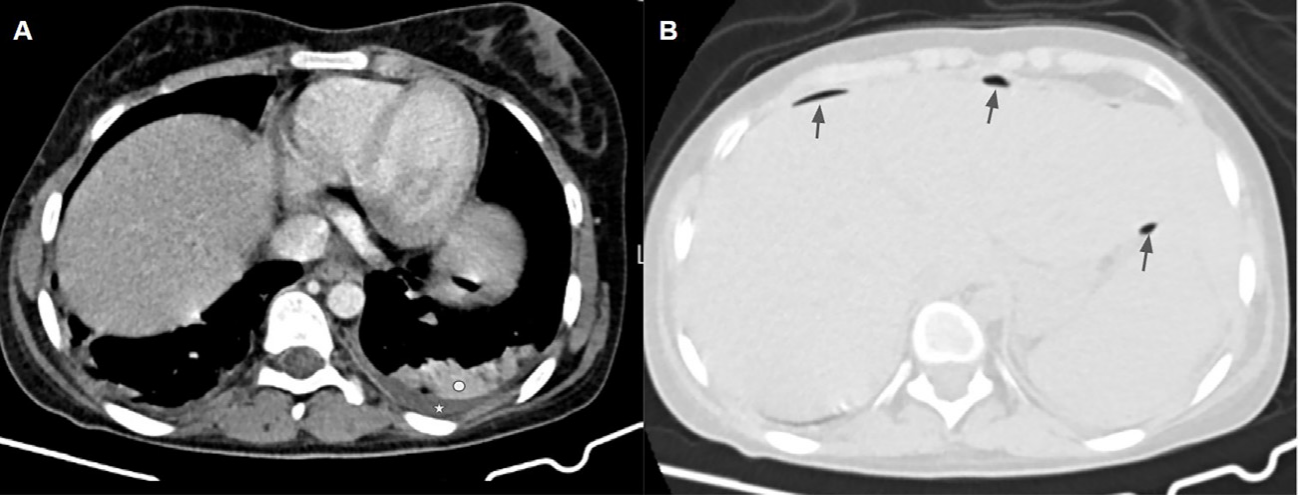
(A) Chest CT scan-Mediastinal window shows left-sided pleural effusion (white star) with adjacent subsegmental consolidation (white circle) in the posterobasal aspect of the left lower lobe. (B) Abdominal CT scan-pulmonary window depicts mild pneumoperitoneum (gray arrows) adjacent to the anterior surface of the liver and spleen.

**Fig. 2:**
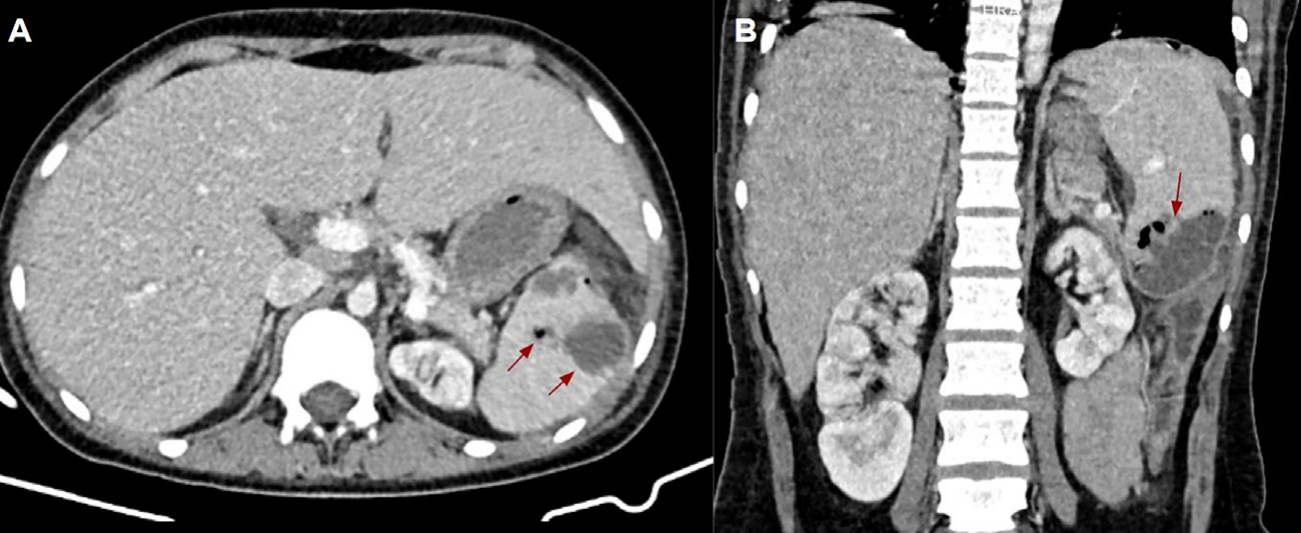
(A and B) Abdominal CT scan with intravenous contrast material demonstrates confluent hypodense collections containing air bubbles (red arrows) in the lower pole of the spleen which is compatible with the diagnosis of splenic abscess in axial and coronal view, respectively.

Laboratory data demonstrated leukocytosis (White blood cell count: 15,000 per microliter) and elevated inflammatory markers (Erythrocyte sedimentation rate: 110 mm/h, C-reactive protein: 90 mg/L). Abdominal ultrasound showed splenomegaly with a splenic diameter of 130 mm craniocaudally and a subcapsular well-defined heterogeneous hypoechoic region in 40 × 29 mm size containing echogenic foci with comet tail artifact in favor of air bubbles at the lower pole of the spleen without vascularity in color-doppler ultrasound. Mild ascites in the pelvic cavity were also found. These findings were mostly compatible with splenic abscess. Transthoracic echocardiography was performed which showed no evidence in favor of endocarditis as a potential origin of splenic abscess. Empirical antibiotic treatment with ceftriaxone and metronidazole was started. The day after admission, an abdominal CT scan with and without intravenous contrast was performed to confirm the diagnosis of splenic abscess (Figs. 1 and 2). In addition, CT investigated the possible complications and assigned the proper treatment to the patient (splenectomy versus percutaneous abscess drainage). The CT scan revealed splenomegaly along with multiple hypodense confluent collections containing air bubbles with total diameters of 42 × 40 × 40 mm in the lower pole of the spleen. Mild perisplenic fluid and air were present close to the external surface of the lower pole of the spleen. There was also evidence of mild pneumoperitoneum and ascites. These findings were highly suggestive of pneumoperitoneum secondary to splenic abscess rupture.

### Surgical management and outcome

Given the aforementioned imaging findings, and no changes in the patient's clinical condition a midline laparotomy was planned and performed, revealing the presence of pus and blood in the peritoneal cavity. After draining these bloody, purulent fluids, and due to damage to most parts of the splenic tissue, the splenectomy was performed without complications. Blood and abscess cultures were sent out, which resulted in negative. Pathological assessment of the extracted spleen reported splenic tissue with multiple subcapsular purulent inflammatory areas and there was no evidence of malignancy. Eventually, immunization against encapsulated bacterial pathogens was prescribed and the patient was discharged 5 days after admission with a suitable clinical condition.

## Discussion

We presented a case that was afflicted by a splenic abscess whose rupture led to pneumoperitoneum. Based on a comprehensive review of the literature, we found only 10 case reports illustrating pneumoperitoneum following the rupture of a splenic abscess [3], [4], [5], [6], [7], [8], [9], [10], [11], [12]. The search strategy used for the purpose of discovering these articles was the utilization of the keywords "Splenic Abscess" AND "Pneumoperitoneum" in the databases of PubMed, Scopus, and Google Scholar Advanced Search.

### Immunosuppression possible role in etiopathogenesis

In the majority of these available cases (70%), an immunocompromised condition was notable. Diabetes mellitus in 4 [[4], [5], [6],^8^] and human immunodeficiency virus (HIV) infection [7], ulcerative colitis under treatment with immunosuppressive drugs and splenic hypoperfusion [10], and malignancy (colon cancer and melanosarcoma) [12] in 1 patient were reported. In the 3 other cases, immunocompromised status was not cited in the patient's past medical histories [3,9,11]. Our case was in line with most reported cases as she had a history of type I diabetes mellitus. This observation could postulate the hypothesis that being immunocompromised predisposes individuals with splenic abscesses to develop pneumoperitoneum as a result of abscess rupture. However, due to small case numbers, this hypothesis requires to be reinforced by further studies.

### Culprit agents

Pneumoperitoneum results from the gas released through fermentation by gas-producing organisms within the splenic abscess [7]. Therefore, similar pathogens must be causative agents in our patient, however, unfortunately, the blood and abscess culture had negative results which may be ascribed to early high-dose antibiotic therapy administration or a technical error. The results of either blood or splenic abscess culture were as follows in reported cases: Positive for *Klebsiella pneumoniae* and anaerobe Prevotella intermedia in pus culture from the splenic abscess [3], positive pus culture for *Enterobacter cloacae* [6], positive pus culture for b-hemolytic Streptococcus, *Escherichia coli* (*E coli*), *Morganella morganii*, and Proteus mirabilis from the perisplenic fluid and positive blood culture for b-hemolytic Streptococcus [7], positive abscess culture for Prevotella intermedia [8], negative blood cultures for any growth of bacteria [9], positive pus culture from abscess for *E coli* and negative blood culture [4], positive abscess aspirate culture for peptostreptococcus species [10], positive pus culture for *E coli* [11]. In addition, the spleen histopathology revealed infiltration of a moderate quantity of Gram-positive bacteria in 1 patient [12] and in 1 case, there was not any data regarding microbial culture [5]. As it is evident from these aforementioned findings, there was no dominant bacterial family or species, but it seems there is a little more proclivity for *E coli* and Prevotella intermedia to be the cause of splenic abscess which could become complicated by pneumoperitoneum.

### Clinical presentation

Apart from typical symptoms of splenic abscess which are classically the triad of fever, left upper quadrant tenderness, and leukocytosis and were noticed in almost all patients in the early course of their disease, pneumoperitoneum following ruptured splenic abscess presents with acute abdomen in 80% of reported cases with signs of diffuse abdominal tenderness, rebound tenderness or involuntary muscle guarding of the abdomen [[3], [4], [5], [6], [7], [8],^11^,^12^]. Our patients did not manifest these symptoms and signs of peritonitis; we presume this observation owes to the very low amount of pneumoperitoneum and peritoneal pus which was not eligible enough to stimulate the peritoneum and if the medical and surgical interventions had been delayed in our case, the acute abdomen would have inevitably ensued.

There is another hypothesis we made for the patient's presentation that we did not find any relevant data in existing case reports and it is the role of the location of splenic abscess in its propensity for rupture. In our case, the location of the abscesses was subcapsular and we suppose that the superficial situation of the splenic abscess makes it more prone to rupture into the peritoneum causing pneumoperitoneum.

### Diagnostic and therapeutic management

The diagnostic approach was quite diverse among case reports. Chest X-rays for pneumoperitoneum and exploratory laparotomy to investigate the cause which showed splenic abscess rupture were utilized in 4 patients [3,5,10,11], abdominal CT scans for both pneumoperitoneum and ruptured splenic abscess in 2 patients [6,7], chest X-ray for pneumoperitoneum and abdominal CT for splenic abscess rupture in 2 patients [4,8], chest X-ray for pneumoperitoneum, abdominal CT firstly did not diagnose splenic abscess but exploratory laparotomy for splenic abscess rupture in 1 patient [9], and abdominal X-ray for pneumoperitoneum and abdominal CT for ruptured splenic abscess in 1 patient [12] were employed. This diversity mainly owes to the clinical condition of the patient as when the patient was clinically stable, a more advanced imaging modality was used. Given our patient, diagnosis of splenic abscess was made by abdominal ultrasound but the diagnosis of its rupture and subsequent pneumoperitoneum was established with abdominal CT scan. Although Chang et al. claim that ultrasound and CT scan have a similar sensitivity for the diagnosis of splenic abscess, CT scan is still considered the gold standard for the diagnosis of splenic abscess [2,13]. We also assume that an abdominal CT scan is the modality of choice for splenic abscess detection particularly when complications such as its rupture and pneumoperitoneum are suspected. Moreover, it assists physicians to plan treatment by delineating the details of the abscess and the topography of the surrounding structures [13].

Except in 1 patient, in all the other case reports, splenectomy was disclosed as the mainstay of treatment (90%) [3,[5], [6], [7], [8], [9], [10], [11], [12]]. It was the same scenario for our patient. Various therapeutic approaches are described in the medical literature for splenic abscess comprising splenectomy, percutaneous abscess drainage, and medical therapy with antibiotics alone; nonetheless, we suppose when perforation of splenic abscess occurs and most of the splenic tissue could not be preserved as it was the case for most reported patients and ours, splenectomy is the most appropriate treatment [1,2].

## Conclusion

Rupture of a splenic abscess is a very rare but potentially life-threatening condition that should be suspected as a cause of pneumoperitoneum, especially in immunocompromised patients. Its diagnosis is confirmed with abdominal CT if the patient is hemodynamically stable, otherwise diagnosed with exploratory laparotomy, and is best treated with broad-spectrum antibiotic therapy and splenectomy.

## Author contributions

EZ was the patient's physician and conceptualized the study. MGM wrote the initial draft and AP and MV revised the manuscript.

## Human rights

All methods were performed in accordance with the relevant guidelines and regulations comprising the Declaration of Helsinki.

## Patient consent

No identifiable information was disclosed in writing this case report; however, written consent was obtained from the patient for publishing his medical data for scientific purposes.
